# *“I beg you…breastfeed the baby, things changed”*: infant feeding experiences among Ugandan mothers living with HIV in the context of evolving guidelines to prevent postnatal transmission

**DOI:** 10.1186/s12889-018-5081-x

**Published:** 2018-01-29

**Authors:** Emma Dunkley, Scholastic Ashaba, Bridget Burns, Kasey O’Neil, Naomi Sanyu, Cecilia Akatukwasa, Jasmine Kastner, Nicole S. Berry, Christina Psaros, Lynn T. Matthews, Angela Kaida

**Affiliations:** 1 Faculty of Health Sciences, Simon Fraser University, Blusson Hall Rm 10522, 8888 University Drive, Burnaby, B.C. V5A 1S6 Canada; 2 Mbarara University of Science and Technology (MUST), Mbarara, Uganda; 3 Massachusetts General Hospital (MGH) Global Health, Boston, MA USA; 4 Behavioral Medicine, Department of Psychiatry, Massachusetts General Hospital (MGH), Boston, MA USA; 5 Division of Infectious Disease, Massachusetts General Hospital (MGH), Boston, MA USA

**Keywords:** HIV-positive women, Breastfeeding, Postnatal transmission, Perinatal transmission, Infant nutrition, HIV, Women, Option B+

## Abstract

**Background:**

For women living with HIV (WLWH) in low- and middle-income countries, World Health Organization (WHO) infant feeding guidelines now recommend exclusive breastfeeding until six months followed by mixed feeding until 24 months, alongside lifelong maternal antiretroviral therapy (ART). These recommendations represent the sixth major revision to WHO infant feeding guidelines since 1992. We explored how WLWH in rural Uganda make infant feeding decisions in light of evolving recommendations.

**Methods:**

We conducted semi-structured interviews with 20 postpartum Ugandan WLWH accessing ART, who reported pregnancy < 2 years prior to recruitment. Interviews were conducted between February–August 2014 with babies born between March 2012–October 2013, over which time, the regional HIV treatment clinic recommended lifelong ART for all pregnant and breastfeeding women (Option B+). Content analysis was used to identify major themes. Infant feeding experiences was an emergent theme. NVivo 10 software was used to organize analyses.

**Results:**

Among 20 women, median age was 33 years [IQR: 28–35], number of livebirths was 3 [IQR: 2–5], years on ART was 2.3 [IQR: 1.5–5.1], and 95% were virally suppressed. Data revealed that women valued opportunities to reduce postnatal transmission. However, women made infant feeding choices that differed from recommendations due to: (1) perception of conflicting recommendations regarding infant feeding; (2) fear of prolonged infant HIV exposure through breastfeeding; and (3) social and structural constraints shaping infant feeding decision-making.

**Conclusions:**

WLWH face layered challenges navigating evolving infant feeding recommendations. Further research is needed to examine guidance and decision-making on infant feeding choices to improve postpartum experiences and outcomes. Improved communication about changes to recommendations is needed for WLWH, their partners, community members, and healthcare providers.

**Electronic supplementary material:**

The online version of this article (10.1186/s12889-018-5081-x) contains supplementary material, which is available to authorized users.

## Background

Globally in 2013, an estimated half of the 240,000 HIV-infected infants acquired HIV through breastfeeding [1]. With implementation of comprehensive infant feeding recommendations and prevention of mother-to-child transmission (PMTCT) practices, including sustained maternal antiretroviral therapy (ART) during pregnancy and postpartum, peri- and post-natal HIV transmission can be reduced from an estimated 15–45% of births [2] to less than 5% in breastfeeding populations [3–6].

Over the last 25 years, World Health Organization (WHO) infant feeding guidelines have evolved [7–13] in response to emerging scientific evidence regarding the risks of postnatal transmission through breastfeeding [3, 7, 14–25]. These risks are balanced against the evidence that not breastfeeding threatens infant health and survival, particularly as HIV treatment becomes increasingly accessible [3, 14–20, 26]. After several updates, WHO infant feeding (2016) [13] and antiretroviral treatment (2013) [12] guidelines recommend exclusive breastfeeding until 6 months, followed by mixed feeding (i.e., the introduction of non-breastmilk liquids and/or solids while breastfeeding) until 24 months, alongside lifelong maternal ART (see Table 1).

**Table 1:** Summary of changes to WHO-led infant feeding guidelines for women living with HIV in resource limited settings

Year	1992	1998	2001	2006	2010	2013	2016
Document Title	WHO/UNICEF Consensus Statement on HIV and Breastfeeding [7]	A Review of HIV Transmission through Breastfeeding [8]	New data on the prevention of mother-to-child transmission of HIV and their policy implications: conclusions and recommendations [9]	HIV and Infant Feeding: update based on the technical consultation held on behalf of the Inter-agency Task Team on the Prevention of HIV Infections in Pregnant Women, Mothers and their infants [10]	Guidelines on HIV and infant feeding 2010: Principles and recommendations for infant feeding in the context of HIV and a summary of evidence [11]	Consolidated guidelines on the use of antiretroviral drugs for treating and preventing HIV infection [12]	Guideline: updates on HIV and infant feeding: the duration of breastfeeding and support from health services to improve feeding practices among mothers living with HIV [13]
Maternal treatment in postnatal period	Not discussed	Not discussed for breastfed infants	Antiretroviral (ARV) prophylaxis recommended, but drug and duration to be decided locally among regimens shown to be effective in Randomized Controlled Trials (RCTs)	ART if required for mother’s health or maternal ARV prophylaxis through 7 days after birth	Lifelong ART if required for mother’s health or ARV prophylaxis through pregnancy until one week after breastfeeding ends	Lifelong ART regardless of CD4 count or clinical stage	Lifelong ART regardless of CD4 count or clinical stage
Infant treatment in postnatal period	Not discussed	ARV prophylaxis (prophylactic efficacy unknown for breastfed infants)	ARV prophylaxis recommended, but drug and duration to be decided locally among regimens shown to be effective in RCTs	ARV prophylaxis for 7 days after birth	If mother not on treatment, daily ARV prophylaxis until 1 week after breastfeeding ends (minimum 4 weeks) or if mother on ART for own health, ARV prophylaxis from birth to 4–6 weeks	ARV prophylaxis from birth to 6 weeks	Not discussed
Infant feeding recommendations for first 6 months	Women should be encouraged to breastfeed regardless of HIV status	1-Replacement Feeding (RF) if possible 2- Breastfeeding with early cessation 3-Treatment of expressed breastmilk 4-Wet nursing	1-RF if AFASS criteria met 2-Exclusive breastfeeding (EBF) for the first few months of life (up to six months)	1- EBF unless AFASS criteria fully satisfied 2-Replacement feeding if AFASS criteria satisfied	EBF	EBF	EBF
All breastfeeding should end	Not discussed	As early as possible	Before 6 months	At 6 months, only if adequate diet is available	At 12 months, only if adequate diet is available	At 12 months, only if adequate diet is available	24 months or beyond (unrestricted) in the context of full support of ART
After EBF ends	Not discussed	Replacement feeding	Replacement feeding	Complementary foods can be introduced at 6 months if replacement feeding is not AFASS	Complementary foods should be introduced at 6 months	Complementary foods should be introduced at 6 months	Complementary foods should be introduced at 6 months
Breastfeeding cessation	Not discussed	Wean as soon as and as quickly as possible	Wean as soon as and as quickly as possible	Wean over a period of 2–3 days up to 2–3 weeks	Wean over a one month period	Wean over a one month period	Wean over a one month period
Counseling	Encourage breastfeeding	Informed decision by mother based on all available options	Informed decision by mother based on all available options	Simplified counseling: only RF and EBF are discussed.	Recommend a single infant feeding option as the standard of care (though maternal and infant treatment may differ)	Recommend a single infant feeding and maternal and infant treatment option as the standard of care	Recommend a single infant feeding and maternal and infant treatment option as the standard of care
Key Evidence	Risk of HIV transmission through breastfeeding was thought to be relatively small [7]	A 1992 Meta-analysis revealed that the risk of HIV transmission through breastmilk was much higher than previously described [14]	Results from a 1999 prospective cohort study showed that EBF in first three months had a much lower risk of HIV transmission than mixed feeding and similar to RF [15]	Evidence reinforced the risk of HIV transmission through mixed feeding and higher rates of child survival through six months of EBF [16, 17]	Evidence of reduced HIV transmission through EBF compared to mixed feeding [18] and results showing reduced transmission risk to < 2% with ART [3, 19] and infant prophylaxis [20] during 6 months of EBF. New evidence showed that abrupt weaning was detrimental to HIV-free child survival [24]	Desire to reduce barriers for pregnant and breastfeeding women to access ART given its demonstrated ability to reduce perinatal HIV transmission [12]	Update to infant feeding guidelines was deemed necessary in light of the introduction of lifelong ART and in response to evidence of the benefits of unrestricted breastfeeding duration among WLWH supported on ART [21–23]

Despite the efficacy of infant feeding recommendations alongside lifelong maternal ART on reducing perinatal and postnatal HIV transmission risk [3–6], WHO guidelines have been criticized for inadequately acknowledging the individual, social, cultural, and health system expectations that influence the infant feeding choices of WLWH [27–29]. Indeed, studies from Uganda and other low and middle-income country (LMIC) settings report that infant feeding practices of a significant proportion of WLWH are inconsistent with national or international recommendations [28, 30–33]. A 2011 study from Uganda found that by three months postpartum, 57% of WLWH practiced exclusive breastfeeding (EBF), when national guidelines recommended EBF until six months [30].

Supporting safe and acceptable infant feeding practices alongside maternal HIV treatment are the linchpins in efforts to eliminate postnatal HIV transmission and promote maternal health. However, little is known about infant feeding experiences of WLWH in the context of evolving recommendations. As a follow-up to finding a high prevalence of depression and challenges with adherence to ART among pregnant and postpartum WLWH in Uganda [34, 35], we conducted qualitative research to explore antenatal and postpartum experiences. Women’s challenges understanding and implementing evolving infant feeding recommendations emerged from the data as an important theme. We share these data in order to inform public health and clinical initiatives aimed at supporting dissemination and uptake of infant feeding recommendations among WLWH.

## Methods

### Study setting

We conducted this study in Mbarara, a town in the western region of Uganda. HIV prevalence in the Mbarara District was 9% among women and 7% among men (2011) [36], and ART coverage among treatment-eligible individuals in Uganda was estimated at 69% (2013) [37]. Approximately 16,000 (13%) infants born to WLWH in Uganda acquired HIV in 2013 [2].

At the time of this study (interviews conducted between February–August 2014, babies born between March 2012–October 2013, and participants reporting on postpartum experiences between March 2012–August 2014), Uganda’s national guidelines for PMTCT and infant feeding [38] recommended that WLWH practice exclusive breastfeeding for 6 months and continue breastfeeding until 12 months alongside the introduction of complementary foods, in accordance with 2010 WHO HIV and Infant Feeding Guidelines [11]. In 2013, with the release of the WHO’s consolidated guidelines on the use of antiretroviral drugs for treating and preventing HIV infection [12], Uganda implemented Option B+, which recommends that all pregnant and breastfeeding WLWH initiate lifelong ART. The regional public sector clinic serving WLWH in Mbarara rolled out Option B+ in January 2010 [39]. Thus, all women in our study were accessing ART for their own health and gave birth after Option B+ implementation.

### Participants

Participants were postpartum WLWH receiving ART from the regional public sector HIV treatment clinic. Participants were recruited from the Uganda AIDS Rural Treatment Outcomes (UARTO) cohort, which followed over 700 men and women living with HIV and initiating ART, with a primary research focus on ART adherence (2005–2015). Female UARTO participants with laboratory-confirmed pregnancy in the two years prior to recruitment were eligible to participate. The primary aim of the study was to explore experiences of antenatal and postpartum depression among WLWH. We used stratified sampling to select eligible UARTO participants across a range of depression scores based on a modified Hopkins Symptoms Checklist (HSCL-16) [34, 40]. The depression severity score was calculated as the mean of the 16 items (possible range 1 to 4), with higher scores indicating greater depression symptom severity. We dichotomized scores into HSCL-16 score ≥ 1.75 indicating “probable depression”, which has been previously used as a positive screen for depression [34, 40] and HSCL-16 score < 1.75. We recruited eligible women across four response patterns to HSCL-16 scores assessed during quarterly UARTO study visits prior to, during, and after the referent pregnancy: (1) HSCL-16 scores falling below 1.75 in the postpartum; (2) HSCL-16 scores rising above 1.75 in the postpartum; (3) stably high (≥1.75) HSCL scores; and (4) stably low (< 1.75) HSCL scores.

This analysis focuses on an emergent theme (infant feeding) that warranted further exploration within a larger study on antenatal and postpartum depression, however, HSCL scores were not used to inform the data analysis.

### Data collection

A female research assistant with training in qualitative research methods contacted potential participants to explain the study purpose. Potential participants had not previously met the research assistant but were familiar with research underway at the clinic. If the woman was interested, the research assistant scheduled an interview at a private location and time chosen by the participant.

The research assistants (NS, CA) conducted individual interviews with WLWH (alone, with no one else present) using a semi-structured interview guide. The guide was developed using guidelines outlined by Miles and Huberman (2002) [41], literature regarding antenatal and postpartum experiences, and input from a multidisciplinary study team with expertise in mental and reproductive health. The interview guide was developed in English, translated into Runyankole (the local language), and back translated. The interview guide (see Additional file 1) explored women’s thoughts, feelings, and experiences prior to, during, and after pregnancy, as a follow-up study to quantitative findings of high rates of depression [34] and mortality [42] around pregnancy in the UARTO cohort.

Interviews were conducted by trained qualitative research assistants fluent in both Runyankole and English, audio-recorded, translated into English, and transcribed. Interviews lasted an average of one hour. No repeat interviews were conducted and transcripts were not returned to participants. Research assistants captured field notes and provided a short summary of each interview. Data saturation was met for the primary objectives of the study. Participants received transport reimbursement of approximately 5 USD.

Socio-demographic and HIV-related clinical data for each participant was collected via linkage to the UARTO cohort database and summarized using standard descriptive statistics.

### Data analysis

Content analysis of transcripts was used to identify participant’s perceptions of the challenges and opportunities experienced during antenatal and postpartum periods. The study team reviewed a subset of transcripts to identify major themes to inform the coding structure, with regular meetings to discuss the coding framework. A codebook was then developed with the analysis team. To ensure consistent understanding and interpretation, two coders (ED and BB) coded the same four interview transcripts. Coding was compared (Kappa statistic = 0.82) [43] and discrepancies were resolved through discussions with the analysis team. The codebook was then updated and the remainder of the interviews were coded independently but with iterative discussions between the coders and the full analysis team. Major and minor themes emerging through coding were identified and discussed with the larger study team. Infant feeding was an emergent theme in women’s descriptions of their experiences. NVivo 10 software was used to organize the data.

## Results

Overall, 42 UARTO participants were eligible to participate in this study. Of 22 eligible women contacted for participation, 20 consented (participation rate = 91%) and were enrolled across the four HSCL-16 response patterns, including four women with falling HSCL-16 scores, six women with rising scores, three women with stably high scores, and seven women with stably low scores.

Of the 20 women interviewed, 14 described an infant feeding experience in response to open questions about challenges and opportunities experienced during the first six months postpartum. Median age of the study participants (*n* = 20) was 33 years (range: 28–35), 70% of women had primary school education or less, median number of living children was 3, and median CD4 cell count was 677 cells/mm^3^. 95% were virally suppressed at the clinical visit closest to interview. Eighteen women (90%) had live births and 2 (10%) experienced other pregnancy outcomes (see Table 2).

**Table 2:** Socio-demographic characteristics of women living with HIV with pregnancy within 2 years prior to qualitative interview (*n* = 20)

Characteristic	Median [IQR] or n (%)
Age (years)	33 [28,35]
Education	
Primary school or lower	14 (70%)
Some secondary school or higher	6 (30%)
Time on ART (years)	2.3 [1.8,5.1]
Number of live births	4 [2,6]
Number of living children	3 [2,4]
Months between pregnancy outcome and interview	15 [7,21]
Most recent CD4 cell count (cells/mm^3^)	677 [440,767]
Suppressed plasma viral load (HIV-1 RNA ≤ 400 copies/ml)	19 (95%)

Among women who discussed infant feeding, 71% (10/14) described a practice inconsistent with WHO recommendations of breastfeeding for at least 12 months, with complementary foods introduced at 6 months. Three participants never breastfed, and the others stopped breastfeeding before 12 months, practiced mixed feeding before six months, or both.

Infant feeding challenges discussed by women are presented according to three major themes: (1) perception of conflicting recommendations regarding infant feeding; (2) fear of prolonged infant HIV exposure through breastfeeding; (3) social and structural constraints shaping infant feeding decision-making.

1. *Perception of conflicting infant feeding recommendations*

Participants reported receiving mixed messages from health care providers, community and family members about optimal infant feeding strategies. Contradictory messages led to confusion, with some women questioning the risks and benefits of mixed feeding, or whether they should breastfeed at all.

One participant who had decided not to breastfeed to avoid the risk of postnatal transmission was told by her partner and a family friend that she was misinformed:

*I told them I will buy for him* [the baby] *milk and… I won’t breastfeed him. …* [T]*he man became mad at me… he had a doctor, his friend, he called me and told me “I beg you, children are these days breastfed. Breastfeed the baby, things changed.”* (Participant #3, Age 40).

Other participants believed that it was safe and/or necessary to introduce non-breastmilk liquids in the first six months in order to overcome perceived low milk supply and increase overall nourishment. Women described practicing mixed feeding prior to 6 months without knowing about the HIV transmission risk:

*…if you are to breastfeed only, the child does not get satisfied and they cry all the time… The child cannot survive on milk alone….I was worried because the child was 2 months and I started to give her what to drink* [non-breastmilk liquids] *but when I came here they told me that I was not supposed to give her anything to drink before she makes 6 months. So I got scared because they told me the gut is not yet stable. She might get an infection and yet you are still breastfeeding her she can easily get ill, so it kept worrying me.* (Participant #2, Age 26).

The safety of mixed feeding was a common source of confusion. This participant chose to stop breastfeeding at 4 months and initiate replacement feeding. Despite being reprimanded by her doctor, she decided not to resume breastfeeding:

*I had stopped* [breastfeeding] *like two weeks, and I came to the clinic and the doctor said, ‘you know what, you have to start today, you have to resume.’ …She even wrote it* [as a prescription]. *I kept quiet and I felt so bad, then I kept wondering, they normally say that these other foods can actually hurt the child, most especially the intestines…does she want me to risk my son? ah ah, I won’t risk.* (Participant #4, Age 31).

Negative interactions with healthcare providers diminished the acceptability of infant feeding recommendations. For instance, one participant described being admonished by her doctor after weaning her child:

*In those months, from one month to 6 months, I kept having worries of breastfeeding him because I … weaned him at 6 months and when I reached…the clinic, the doctor abused me. He said no, “…children are supposed to breastfeed for a year.” I told him …I already weaned. He said “you did bad* [stopping breastfeeding]*…don’t you see he had just started* [breastfeeding]*.”* (Participant #3, Age 40)

2) *Fear of prolonged infant HIV exposure through breastfeeding*

Women’s awareness or direct experience that HIV can be transmitted through breastmilk contributed to confusion and distrust of recommendations to breastfeed, particularly among women who had lost previous children to HIV or who were caring for children living with HIV. This participant describes the reason she stopped breastfeeding:

*Participant (P): Ehh my friend I couldn’t wait* [to stop breastfeeding]*.*

*Interviewer (I): You couldn’t wait, was that a doctor’s recommendation?*

*P: No, because for them they told us one year. … I was like, ‘one year?’... I don’t want to take risks because … I already know what it means* [to have a child with HIV]*, so I said no, let me stop* [breastfeeding] *and find how to look after him.* (Participant #4, Age 31).

Most participants who chose to breastfeed described the experience as an ongoing source of worry as it extended the period of postnatal HIV exposure [12].

*I: So in those six months you were breastfeeding him, what were your thoughts at that time?*

*P: I used to pray to God to be the one to protect him. …So that he does not get infected with HIV … because of breastfeeding….* (Participant #10, Age 28).

Breastfeeding mothers also described the increased burden of infant testing and the need for ongoing infant prophylaxis [44]. As one participant explains:

*Let’s say if you continue breastfeeding, they continue testing and testing* [for HIV]. *But for this one* [her child who stopped breastfeeding at 6 months]*, they will test him again when he is one and a half years…that will be the last test. But when you are still breastfeeding, they keep testing and testing.* (Participant #4, Age 31).

Many women described feeling that they could only relax after their child’s last HIV test. One participant who chose to breastfeed describes the experience of testing her child for HIV:

*I: When they told you the first results were fine, how did you feel?*

*P: I became somehow strong but I knew maybe it is not yet visible…since I am breastfeeding and…I can’t sustain myself* [manage] *to stop breastfeeding her.*

*I: …you went a second time, how did you feel?*

*P: I became a bit strong but not so strong because she was still breastfeeding, I said I will be strong after getting the final results when she has weaned.* (Participant #20, Age 35).

3) *Social and structural constraints shaping women’s infant feeding decision-making*

The preferences and expectations of partners and family, the experiences of other WLWH in the community, and economic constraints influenced participants’ decisions and experiences of infant feeding. This participant describes her partner’s influence on her decision to breastfeed:

*…when I produced him* [gave birth]*, immediately I never breastfeed him. The man begged me, “please breastfeed the baby.”* (Participant #3, Age 40).

Expectations from family about breastfeeding norms were also important drivers of infant feeding practices. As one participant explains:

*There is this woman in the village that…will always come to the health facility but she does not want anyone in the family to know* [that she is living with HIV]*. If she wants to wean the child the mother-in-law says no, and she continues to breastfeed.* (Participant #17, age 31).

Economic circumstances also affected women’s infant feeding choices. As one participant described:

*They say if you…can manage you should buy all the feeds to look after him, including baby formula, yet I did not have the money.* (Participant #10, Age 28).

Beyond the cost of formula, women’s knowledge of the financial and emotional burden of caring for a child living with HIV was a significant consideration:

*What prevented me most from breastfeeding was HIV. When I see a child with HIV I feel so sad and when you do not have money and add on a child that is sick* [HIV-positive]*, you may fail to raise them. Because an HIV-positive child needs a lot of care… When you see a sick child in the village you pray for them to die so that they can rest.* (Participant #17, Age 31).

## Discussion

Our findings revealed infant feeding as a substantial challenge and source of worry for postpartum WLWH. Participants described infant feeding practices that ran counter to recommendations, particularly challenges initiating breastfeeding, exclusive breastfeeding in months 0–6, and mixed feeding during months 6–12 and beyond. Data revealed women’s uncertainty as they navigated powerful decisions about how to nourish their child, prevent HIV transmission, manage the stress of prolonged infant HIV exposure while breastfeeding, and incorporate social and structural factors affecting infant feeding choices.

Participant narratives reveal confusion and mistrust about the recommendation to initiate mixed feeding at 6 months. The strong emphasis on the importance of exclusive breastfeeding in months 0–6 and earlier WHO (2001, 2006) recommendations to avoid mixed feeding at any time in the postnatal period may have contributed to women’s distrust of providers’ instructions, particularly among older women who may have been exposed to different recommendations in previous pregnancies. The perceived insufficiency of exclusive breastfeeding was also reflected in women’s infant feeding decisions. Several participants felt that breastfeeding alone was an insufficient source of infant nutrition, particularly in the context of maternal undernutrition and food insecurity [45, 46]. This led some participants to question their infant feeding choices or to adopt an infant feeding approach that, if a mother was not virally suppressed, may have inadvertently increased the infant’s risk of acquiring HIV. Underlying this was a lack of knowledge or trust that HIV transmission increases when non-breastmilk foods and liquids are introduced before 6 months of age. When challenged on their infant feeding choices by a health care provider, many women expressed that they felt they were practicing the ‘right’ infant feeding method – as a result of a previous pregnancy, personal experience, or the recommendations of friends or family. Women’s descriptions of health care provider interactions suggest a need for greater consideration of women’s prior experiences, beliefs and expectations when counseling on best infant feeding practices.

This study demonstrates that the fear of postnatal HIV transmission remains at the forefront of WLWH’s postpartum experiences and that prolonged infant HIV exposure while breastfeeding is not readily accepted. Participants discussed their knowledge of HIV transmission through breastmilk, but none discussed how viral suppression reduced postnatal HIV transmission risks, or if it impacted their infant feeding decisions. Women described their desire to cease breastfeeding and receive the last test to know an infant’s HIV status. This finding is particularly relevant given that WHO 2016 HIV and Infant Feeding Guidelines [13] state that breastfeeding duration should not be restricted, i.e., up to 24 months or beyond, for WLWH accessing supportive care for ART adherence. These recommendations reflect evidence that continued breastfeeding improves HIV-free survival among HIV-exposed infants in LMICs, however, this study shows how stressful it can be for a woman accessing ART to breastfeed. These findings further suggest that additional supports are required to alleviate concerns about breastfeeding duration, infant health and survival, and infant HIV exposure.

Infant feeding practices occur in the context of past childbearing and rearing, experiences of other WLWH, the influence of family and partners, and economic constraints. While most women in this setting cannot meet criteria to recommend replacement feeding, some women described formula feeding as a more desirable, albeit largely unaffordable, option and expressed guilt about their inability to provide formula. In contrast, none of the women interviewed discussed the benefits of breastfeeding for infant health and survival. Consistent with research described elsewhere [47–49], health care providers, partners, and other family members play an important role in a WLWH’s infant feeding choices. These findings highlight a need to examine how best to communicate infant feeding guidelines and support women to make informed decisions, particularly with changing recommendations.

### Limitations

This qualitative study includes a small sample size and is not meant to be generalized. Infant feeding was an emergent theme in an interview focused on postpartum experiences more generally, without specific questions to probe issues and experiences related to infant feeding. Furthermore, with 95% of participants virally suppressed with regular access to health care services including ART and counseling, the experiences of women in our study are not representative of the situation for women in the rest of Western Uganda, where a recent study in Kabarole District found 51% maternal ART adherence at 6 months postpartum after the rollout of Option B+ [50]. Given that our participants’ experiences with counseling and care are likely better than for most, our findings likely describe a best-case scenario regarding challenges navigating evolving infant feeding recommendations.

### Implications and recommendations

While infant feeding guidance is present within national and sub-national policies, the findings of this study suggest that further research is needed to explore how health care systems can best support WLWH with infant feeding in the context of evolving postnatal transmission recommendations. As the 2016 guidelines recommend breastfeeding for WLWH up to 24 months and beyond, there may be additional confusion and concerns among healthcare providers and patients.

Specific recommendations include:

1. **Research on communication of infant feeding recommendations from health care workers to WLWH:** In this analysis, women expressed uncertainty and distrust about infant feeding recommendations, which were compounded by negative interactions with health care providers. Additional research is needed to understand how infant feeding recommendations are communicated to WLWH as guidelines change and to identify where improvements can be made. Health care providers providing infant feeding counseling must be confident in the accuracy of their recommendations and must be able to effectively communicate infant feeding options to WLWH, particularly the benefits of breastfeeding while virally suppressed. Moreover, infant feeding counseling must acknowledge and incorporate the social and structural pressures facing WLWH, with health care providers aiming to support WLWH as they make personal decisions about infant feeding.
2. **Clear and supportive guidance on infant feeding choices and practices for WLWH, their partners, and families.** WLWH, and particularly those with previous pregnancy experiences, require focused information and support regarding infant feeding, particularly the diminished risks of HIV transmission while virally suppressed and the benefits of breastfeeding. In addition to clinical settings, it is important that this information be accessible in community settings, with uptake of innovative delivery mechanisms, including through peer support [51].
  Study results and previous evidence point to the need for family and partner support with infant feeding [31]. Public health initiatives to promote greater awareness and acceptance of recommended infant feeding practices by partners and family members may increase women’s confidence in their infant feeding choices, particularly as guidelines change.

## Conclusions

For WLWH, infant feeding can be a complex and layered challenge. Globally, advocacy efforts have called for a balance between the rigidity of recommendations based on emerging scientific evidence and the reality of women’s social, economic, cultural and emotional circumstances. Sustained ART use is associated with increased fertility intention [52] and pregnancy incidence [53] among WLWH, and care providers and policy makers must extend a continuum of support before, during, and *after* pregnancy. Participants in this study were nearly all virally suppressed, with consistent access to ART, medical care and counseling. Even in such a setting, our findings reveal that recommendations to prevent postnatal HIV transmission may not readily translate into practice, thus compromising maternal and child health and survival. This analysis suggests that WLWH’s choice and experience of infant feeding needs attention, to decrease maternal stress in the postpartum period, confidently implement infant feeding choices, and benefit from the reduced HIV transmission risks accompanying sustained use of ART and viral suppression. Particularly, as infant feeding recommendations shift to optimize maternal and child health and survival, health care providers must ground care in women’s current circumstances and past experiences to see these benefits realized.

## Additional file

Additional file 1: PPD Interview Guide. (PDF 481 kb)

## Abbreviations

AFASS: Acceptable feasible affordable sustainable and safe
ART: Antiretroviral therapy
ARV: Antiretroviral
EBF: Exclusive breastfeeding
HSCL: Hopkins symptoms checklist
LMICs: Low- and middle-income countries
PMTCT: Prevention of mother-to-child transmission
RCT: Randomized controlled trial
RF: Replacement feeding
UARTO: Uganda AIDS rural treatment outcomes
WHO: World Health Organization
